# Complications Associated with Permanent Internal Jugular Hemodialysis Catheter: A Retrospective Study

**DOI:** 10.7759/cureus.4521

**Published:** 2019-04-22

**Authors:** Syed Muhammad Shahnawaz Hyder, Junaid Iqbal, Irfan Amjad Lutfi, Muhammad K Shazlee, Kamran Hamid, Sadia Rashid

**Affiliations:** 1 Radiology, Dr Ziauddin University Hospital, Karachi, PAK; 2 Radiology, Shaheed Mohtarma Benazir Bhutto Accident Emergency & Trauma Center, Civil Hospital, Karachi, PAK; 3 Radiology, The Indus Hospital, Karachi, PAK

**Keywords:** vascular access, permcath, complication, hemodialysis, end-stage-renal-disease

## Abstract

Introduction

A significant increase in patients with end-stage renal disease has been observed currently in our community. Kidney transplantation is the most promising cure but the problem is that large numbers of people are not good candidates for transplantation. Hemodialysis is the next appropriate medication for such patients and for patients with end-stage renal disease, who have no chance for transplantation. Morbidity and mortality are the consequences of vascular access complications. Local data related to the complication rate of permanent hemodialysis catheters is not available. The current study examines the complication rate in people due to permanent intrajugular hemodialysis catheterization.

Materials and methods

The study has been conducted in Dr Ziauddin University Hospital, Karachi. The dataset consists of 212 patients who had gone through jugular catheterizations for hemodialysis at this hospital from the year 2014 to the year 2015. A descriptive method has been chosen for obtaining appropriate results. Complications have also been categorized as early or late.

Results

Complications have been detected in around 24% of the patients from the dataset. Among these complications, infection has the highest percentage (around 13%) while 4% percent of patients have a failed puncture. The others have venous thrombosis, catheter thrombosis, hematoma, wrong canulation, and hemothorax and pneumothorax problems.

Conclusion

The study concludes that the placement of a permanent hemodialysis catheter in the internal jugular vein has a low complication rate. In addition, the method is safe and easy. So, it can be said that the internal jugular vein is a reliable and preferred route for hemodialysis catheterization.

## Introduction

Nowadays, in our community, substantial numbers of people are suffering from end-stage renal disease. The most long-lasting cure for this serious condition is kidney transplantation. But transplantation is not suitable for around 60% of the people [1]. In such cases, hemodialysis is the next optimal option [2-3]. Hemodialysis requires good vascular access sites with at least 350 ml/min of blood flow. In the absence of good vascular access, the quality of hemodialysis reduces [2].

The major source of high morbidity and mortality are vascular access complications [2]. Half of the hospitalization costs of patients having end-stage renal disease are related to vascular complications [4]. Due to its long-lasting access, an arteriovenous fistula is the most appropriate technique of vascular access. But various complications are linked with it [5-6]. These include thrombosis, hand ischemia, edema, bleeding, aneurysm, carpal tunnel syndrome, and infection [2]. Infection, stenosis of the jugular vein, and thrombosis are also common complications of permanent intrajugular hemodialysis catheterization [7].

The best access to perform the process of hemodialysis is an arteriovenous fistula (AVF). It has a low complication rate as well as long-term usage. In patients where the formation of an AVF is not possible or for those who require dialysis during the maturation of the AVF, a hemodialysis catheter in the central arteries is an appropriate option [8].

Despite all the complications, using a dialysis catheter is still very common. Around 63% of patients used a catheter for vascular access for their first dialysis treatment in the USA per the annual report of the United States Renal Data System (USRDS). An AVF for vascular access for the first dialysis has been used by only 16% of the patients. Eighty-one percent of patients are waiting for AVF maturation and, till then, are using a dialysis catheter, considering it the only method of vascular access [9-10].

Hemodialysis catheters were initially developed for short- and medium-term hemodialysis access to cater to the AVF maturing period required for the permanent form of access. The first report demonstrating the effectiveness of hemodialysis catheters was published by Schwab et al. in 1988 [11]. In the late 90s, there has been increased reliance upon these semi‐permanent catheters as a means of permanent access in patients on chronic hemodialysis [12]. The reasons for this include the ease of insertion, the increasing proportion of elderly and diabetic patients with vessels for which AVF is unsuitable, and the choice of the patient [11].

The local datasets related to complications as a consequence of permanent hemodialysis catheters are not available. This work examines the complication rate of permanent intrajugular hemodialysis catheterization in our population.

## Materials and methods

This study has analyzed a data set of 212 patients who went through internal jugular catheterization for hemodialysis at Dr. Ziauddin University Hospital in the years 2014 and 2015. Among these, the ratio of men to women is 67 to 33. A descriptive method has been used for obtaining results. Complications have been divided into two classes: early and late. The Quinton double lumen polyurethane catheter (Quinton, Seattle, WA, USA) was used in all cases. All were inserted percutaneously by the consultant interventional radiologists with a minimum of two years experience. Insertions were always performed using complete aseptic precautions. The catheter was dressed with sterile gauze and the dressings were changed at least three times a week. Each catheter was left in only as long as it was required or for a maximum period of 12-16 weeks, provided there were no complications.

## Results

Baseline characteristics

Out of a total of 212 patients, the mean age of the patients was 57.67±12.74 years (range 21-90 years). Males were predominantly higher 142 (67%) than females 70 (33%). The mean duration of the catheter was 79.6±37.35 days (range 29-122 days).

Complications

It has been observed that 58 patients out of 212 faced complications as a result of treatment. Nineteen patients faced early complications and 39 patients faced late complications. In these patients, the most common complication was infection. The other complications include failed puncture, venous thrombosis, catheter thrombosis, and hematoma. Wrong canulation, haemothorax, and pneumothorax were also observed.

Early Complications

**Figure 1:**
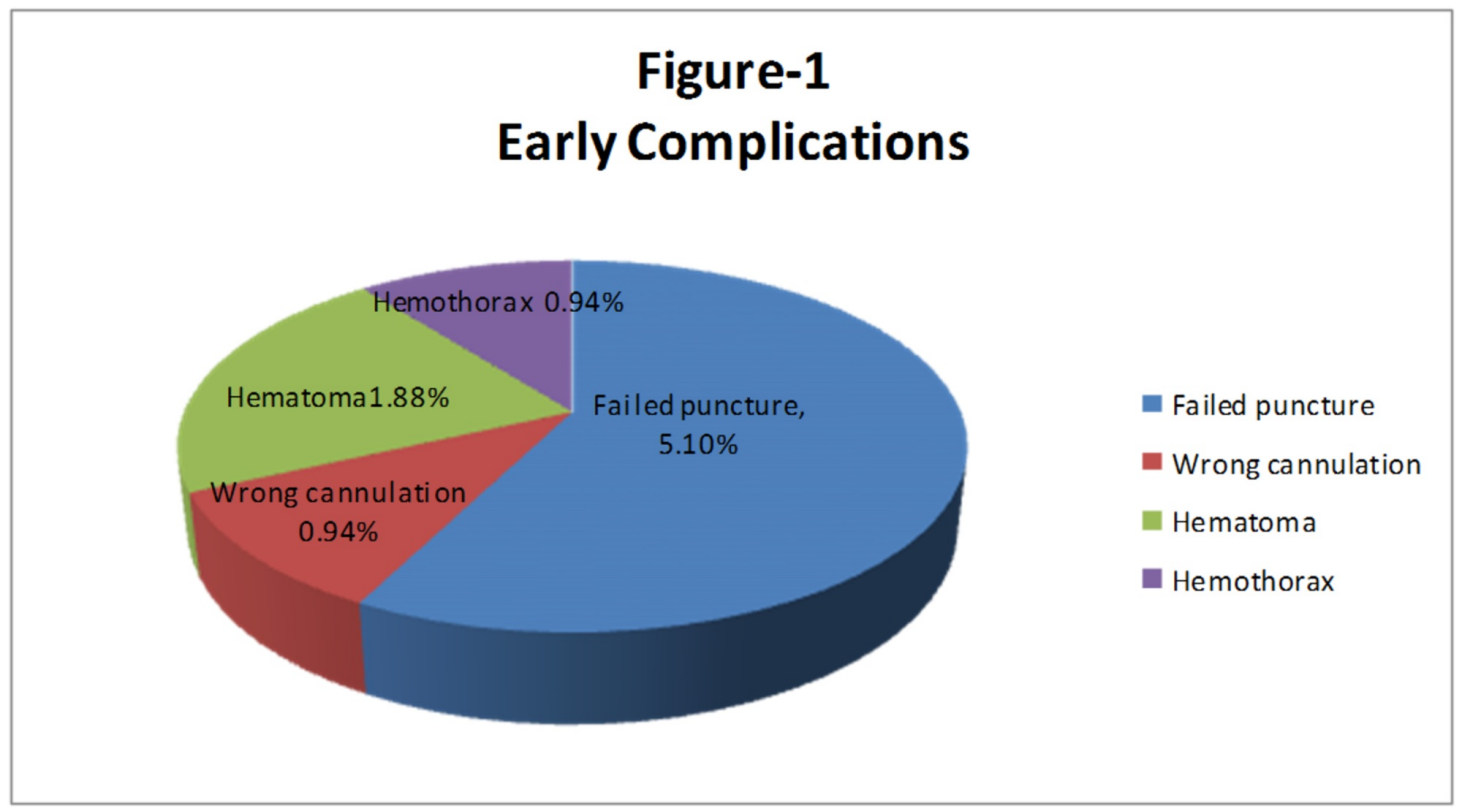
Early complications

The early complications are shown in Figure 1.

Failure to puncture: In 11 (5.1%) patients, we were unable to puncture the internal jugular vein even on the opposite side. The procedure was then postponed and planned for other types of access such as femoral access.

Accidental puncture: In two (0.94%) patients, there was accidental cannulation. In one patient, there was a puncture of the subclavian artery while in the other patient, there was a puncture of the common carotid artery. The needle was removed in both cases; no complication was seen in the subclavian artery puncture while in the carotid artery puncture, there was a small hematoma seen, compression was applied, and the hematoma resolved on a follow-up scan.

Hematoma formation: In four (1.88%) patients, there was small, local hematoma formation at the site of puncture, compression was applied, and the hematoma resolved on follow-up scans. In all four patients, the successful placement of the catheter was done from the opposite side.

Haemothorax: In two (0.94%) patients, just after the procedure, there was opacification in the right hemithorax, which on aspiration turned out to be blood. Chest tube drainage was arranged urgently. The patient remained stable and the chest tube was removed after one week.

Late Complications

**Figure 2:**
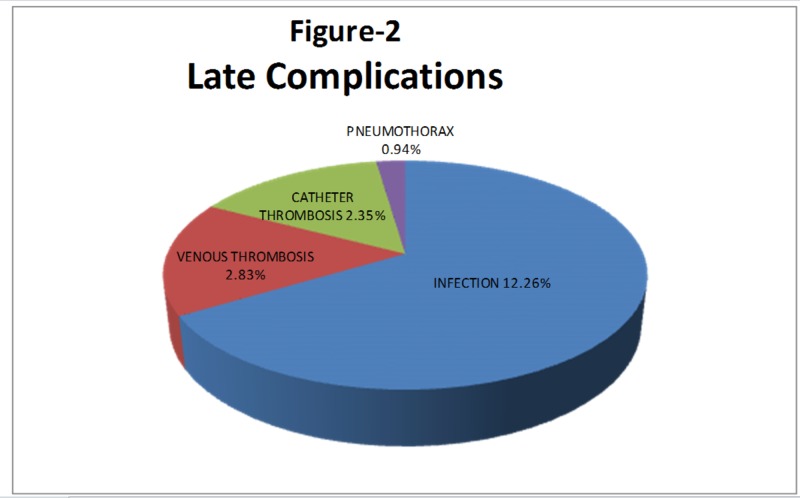
Late complications

The late complications are shown in Figure 2.

Infection: This is the most common complication and occurred in 26 (12.26%) patients. Patients presented with pain, fever, redness, and small pus discharge at the site of catheter insertion. Antibiotics were given to all patients; 14 patients responded while 12 patients didn’t. The catheter was removed in all 12 patients who didn’t respond to the antibiotic trial. The catheter tip was sent for culture and in nine patients, the culture turned out to be Staphylococcus aureus while in three patients, it turned out to be Pseudomonas.

Venous thrombosis: Six (2.83%) patients developed thrombosis of the internal jugular vein. Patients presented with facial swelling. A CT scan was performed and there was thrombosis of the internal jugular vein. The catheter was removed and anticoagulant treatment was given. The patients responded well.

Catheter thrombosis: Five (2.35%) patients developed thrombosis of the catheter. All five patients presented with unsuccessful hemodialysis within one month of placement of the catheter. In all patients, we used streptokinase to open the catheter; the trial was successful in three patients while in two patients, the catheter did not open by the use of streptokinase, so we replaced the catheter.

Pneumothorax: This was seen in two (0.94%) patients. In one patient, there was small pneumothorax and no further intervention required while in one patient, the pneumothorax was large and the patient felt shortness of breath. In this patient, the chest tube was placed, he responded well, and the chest tube was removed after eight days.

Comparison of complications with general characteristics

**Figure 3:**
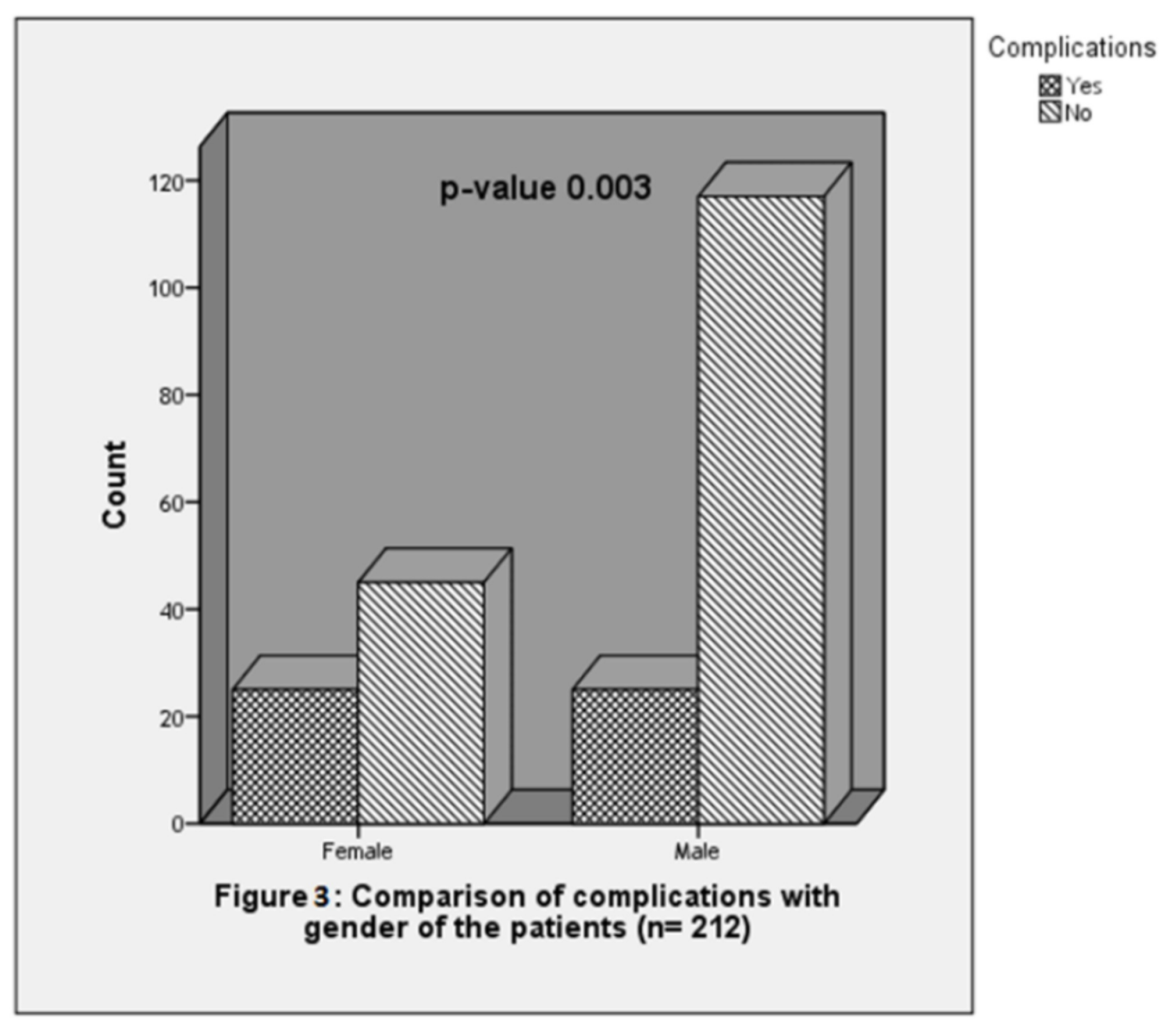
Comparison of complications with gender of the patients (n=212)

Complications were observed to be significantly higher among females (25 or 25.7%) as compared to males (25 or 17.6%); p-value 0.003 (Figure 3). The mean age of the patients was observed higher in patients with infections (67.64±9.18 years) followed by catheter thrombosis (60.2±16.3 years) and wrong canulation (59±10.81 years). The mean duration of the catheter was observed to be higher among patients with venous thrombosis (89.4±40.4 days) followed by infection (84.7±37.91 days) and infection (70±40.98 days). The comparison of complications with respect to general characteristics is shown in detail in Table 1.

**Table 1:** Comparison of complications with general characteristics of the patients (n=212)

Table 1: Comparison of complications with general characteristics of the patients (n=212)									
Variables	Failed Puncture (n=8)	Wrong Canulation (n=2)	Hematoma (n=4)	Hemothorax (n=2)	Infection (n=27)	Venous Thrombosis (n=7)	Cathethor Thrombosis (n=6)	Pneumothorax (n=2)	Total
	n (%)	n (%)	n (%)	n (%)	n (%)	n (%)	n (%)	n (%)	
Age*, years	56.01 ±8.22	59.00±10.81	57.85±2.73	57.80±12.84	67.64±9.18	54.17±15.51	60.20±16.3	57.70±4.67	
≤60	5 (62.5)	0 (0)	2 (50)	0 (0)	13 (48.14)	4 (57.14)	3 (50)	1 (50)	28 (48.3)
>60	3 (37.5)	2 (100)	2 (50)	2 (100)	14 (51.85)	3 (42.85)	3 (50)	1 (50)	30 (51.7)
Gender									
Male	3 (37.5)	1 (50)	2 (50)	0 (0)	12 (44.4)	5 (71.4)	1 (16.7)	1 (50)	25 (43.1)
Female	5 (62.5)	1 (50)	2 (50)	2 (100)	15 (55.6)	2 (28.5)	5 (83.3)	1 (50)	33 (56.9)
Duration of symptoms*, days	70 ±40.98	58.5 ±41.71	48.5 ±28.58	31.5 ±3.53	84.7 ±37.91	89.4 ±40.4	43.2 ±21.6	58.5 ±41.7	
≤90	5 (62.5)	2 (100)	3 (75)	2 (100)	10 (37)	3 (42.85)	6 (100)	2 (100)	33 (56.9)
>90	3 (37.5)	0 (0)	1 (25)	0 (0)	17 (63)	4 (57.14)	0 (0)	0 (0)	25 (43.1)
*mean ±SD									

The association of gender was found significant with hemothorax (p-value 0.43), infection (p-value 0.014), and catheter thrombosis (p-value 0.008). A substantial difference was also perceived in the duration of catheter and infection (p-value 0.015) whereas an insignificant difference was observed in age and complications (p-value>0.05).

## Discussion

Hemodialysis is the preferred treatment for end-stage renal disease. In order to perform good quality dialysis, sufficient access to blood flow is required by hemodialysis. For access to blood with sufficient flow, the preferred method is to form an AV fistula in which anastomosis is made between the artery and vein.

After the formation of AV fistulae, it takes almost five to six weeks for the maturation of the AV fistulae to be used for hemodialysis [13]. In some cases, the deficiency of appropriate vessels for AVF or sometimes because of some complications, it is not possible to perform dialysis through the AVF. In such conditions, central venous catheters are used. Permcaths cause less endothelial damage because they are made of ​​softer material. Consequently, less long-term complications have been witnessed [14]. The internal jugular vein is considered to be the best location for the placement of these catheters [15].

In our study, complications occurred in 22.6% of the patients. The complications usually appeared late. The most common complication was infection, and this is most likely due to the catheters being left in for a long time. The most common organism is Staphylococcus aureus. Venous thrombosis and catheter thrombosis are the next most common complications. Again, these are in the category of late complications, most likely due to the catheters being left in for a long time. The common early complications at the time of the procedure were a failure to puncture the internal jugular vein and local hematoma formation; these could be due to repeated catheterizations. To reduce the rate of complications, it is recommended that the catheterization should be performed only by trained radiologists, under a completely aseptic technique, and the catheter should be removed as soon as the need is over.

In various studies, it was concluded that the complication rate of internal jugular vein catheterization was low as compared to other access, and the internal jugular vein is the ideal position for hemodialysis catheter placement. Central venous catheters placed in the femoral vein have a higher rate of infection and lower limb deep venous thrombosis [16]. Allen et al. [17] mentioned an overall thrombosis rate of 38% among patients with peripherally inserted central catheters.

According to Cimochowski et al. [18], as a result of the insertion of dialysis catheters, a 50% stenosis rate of the subclavian and a 0% stenosis rate of the internal jugular vein has been detected. Bambauer et al., in a retrospective study [19], concluded that the overall complication rate for a subclavian puncture is almost twice that of an internal jugular puncture. The highest complication rates for both vascular access were infections, which were observed in 19.5% of subclavian and 10% of internal jugular catheterization.

## Conclusions

From the results presented here, it can be concluded that the complication rate was very low in the placement of the permanent hemodialysis catheter in the internal jugular vein. Moreover, it is an easy and safe option. Therefore, it can be said that the internal jugular vein can be preferred for hemodialysis catheterization, as it is reliable.
